# Natural product-likeness score revisited: an open-source, open-data implementation

**DOI:** 10.1186/1471-2105-13-106

**Published:** 2012-05-20

**Authors:** Kalai Vanii Jayaseelan, Pablo Moreno, Andreas Truszkowski, Peter Ertl, Christoph Steinbeck

**Affiliations:** 1 Chemoinformatics and Metabolism, European Bioinformatics Institute (EBI), , UK; 2 Institute for Bioinformatics and Cheminformatics, University of Applied Sciences of Gelsenkirchen, Germany; 3 Novartis Institutes for BioMedical Research, , Switzerland

## Abstract

**Background:**

Natural product-likeness of a molecule, i.e. similarity of this molecule to the structure space covered by natural products, is a useful criterion in screening compound libraries and in designing new lead compounds. A closed source implementation of a natural product-likeness score, that finds its application in virtual screening, library design and compound selection, has been previously reported by one of us. In this note, we report an open-source and open-data re-implementation of this scoring system, illustrate its efficiency in ranking small molecules for natural product likeness and discuss its potential applications.

**Results:**

The Natural-Product-Likeness scoring system is implemented as Taverna 2.2 workflows, and is available under Creative Commons Attribution-Share Alike 3.0 Unported License at http://www.myexperiment.org/packs/183.html. It is also available for download as executable standalone java package from http://sourceforge.net/projects/np-likeness/under Academic Free License.

**Conclusions:**

Our open-source, open-data Natural-Product-Likeness scoring system can be used as a filter for metabolites in Computer Assisted Structure Elucidation or to select natural-product-like molecules from molecular libraries for the use as leads in drug discovery.

## Background

Natural products (NPs) are small molecules synthesised by living organisms. In drug discovery, the class of NPs termed secondary metabolites that are involved in defence or signalling, are of particular importance because they were optimised during evolution to have effective interactions with biological receptors. They are therefore good starting points for designing new drugs [1]. Hence, Natural Product-likeness (NP-likeness) of a chemical structure can serve as a criteria in lead compound selection and in designing novel drugs [1]. In order to estimate NP-likeness of a molecule, prior knowledge such as physicochemical and structural properties of existing natural products have to be captured. In this work, we focus only on identifying structural features typical of natural products, and based on their presence, rank molecules of interest according to their NP-likeness.

## Methods

CDK-Taverna version 2[2,3] is an open-source Java tool kit to perform cheminformatics tasks, making use of the pipelining technology offered by Taverna version 2.2[4], an open-source workflow management system. The CDK-Taverna 2 plug-in is based on the Chemistry Development Kit (CDK) [5,6] and few other open source Java libraries. The individual components required to score a small molecule for NP-likeness are implemented as CDK-Taverna workflows to be used intuitively by users without programming background. Source code for the CDK-Taverna 2 workers is freely available at https://sourceforge.net/projects/cdktaverna2/.

The scorer is also available as standalone Java ARchive (JAR) package to be used as a library component in stand-alone or web applications. The standalone JAR and the source code is freely available for download at http://sourceforge.net/projects/np-likeness/.

### Integration of NP-Likeness scorer components with CDK-Taverna 2.2

CDK-Taverna 2 [2,3] has drag and drop components (workers) to build cheminformatics workflows ranging from parsing a molecule file via fingerprinting and clustering to more advanced tasks such as reaction enumeration. The full features of the CDK-Taverna 2.0 plug-in, its installation procedure and example workflows are available at http://cdk-taverna-2.ts-concepts.de/wiki/. CDK-Taverna 2 provides a set of workers commonly used in cheminformatics workflows. To provide additional functionality, individual components such as those required to score a small molecule for NP-likeness are bundled as sub-packages within the existing CDK-Taverna2 plug-in. The NP-likeness sub-packages comprise workers for molecule curation, fragment generation and fragment scoring; all of which can readily be integrated into other data analysis workflows.

### Components for molecule curation

Before being evaluated for NP-likeness, molecules have to be pre-processed to remove small disconnected fragments like counter-ions and fragments containing metallic elements. In previous study [1] commercial tools such as PipelinePilot and Molinspiration [7,8] were used to standardise molecules. These curation workers are now implemented in an open manner within the CDK-Taverna 2.0 plug-in and available under the folder “Molecule curation”. To start with, worker checks for the disconnected parts in the molecule. If such are found, the user has an option of configuring the minimum atom-count for a fragment to be retained. As suggested by *Ertl et al*.[1], the default minimum atom-count cut-off is set to 6 and so, unless modified, disconnected fragments with less than 6 atoms will be removed from the molecule. The worker filters molecules, removing those that contain elements other than C, H, N, O, P, S, F, Cl, Br, I, As, Se or B. As another standardisation step, deglycosylation is needed to remove sugar moieties from the molecules. worker identifies all the sugar moieties in the structure and remove the ones that are linked by glycosidic bond to the scaffold. This is done in order to retain core structural features that are more typical of natural products and to omit features like sugar moieties that are less distinctive, albeit commonly present in natural products. Removal of sugars is not expected to improve the score but to facilitate classifications based only on chemically interesting structural features.

**Figure 1:**
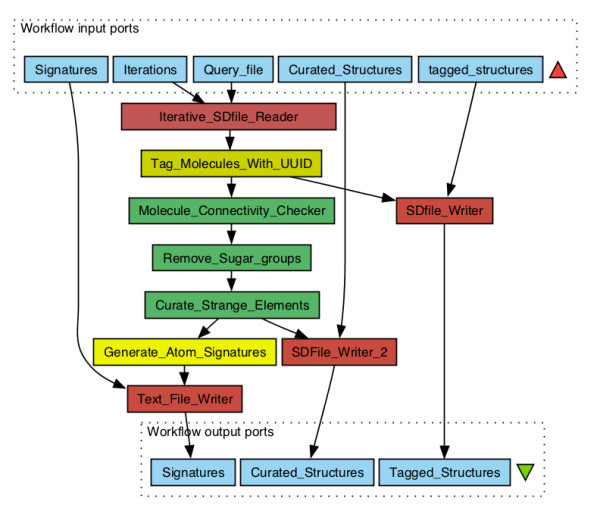
**Molecule curation and atom signature generation workflow.** This workflow takes input of compounds and performs curation and atom signature generation for every compound structure. takes input of compounds () in Structure Data Format (SDF) file. The input can be a single SDF file or list of files. The number of compounds to be read and passed down the workflow for each iteration is specified using the port . As soon as the compounds are read, the worker tags every compound with a UUID. This step helps in keeping track of compounds until the end of the scoring process. As a first step in the curation process, the checks for the connectedness of the atoms in the compound structure. This step removes counter ions and other small disconnected fragments. worker removes linear and ring sugars from the compound structures. Finally, the compound structures are checked for the presence of elements other than non-metals, and if present the structures are discarded by the worker. The curated molecules are consumed by the worker to generate atom signatures for every atom in the compound structure. The generated atom signatures are written out to a text file () for re-use. At any step of the process, the curated and discarded structures can be written out to an SDF file. In this workflow, initially tagged compounds () and fully curated compounds () are written out to SDF files. This workflow is available for free download at http://www.myexperiment.org/workﬂows/2120.html.

An example workflow that makes use of all the curation workers is depicted in Figure 1. The workflow takes Structure Data Format (SDF) file of molecules from the user as input. As soon as the molecules are read, they are assigned an Universal Unique IDentifier (UUID) before entering the curation step. The UUID tagging is done in order to keep track of molecule fragments generated upon curation. For example, when a sugar ring connecting two different scaffolds of a molecule is removed the molecule is split into two fragments. These fragments will have the same UUID of the parent molecule and will be tracked as single molecule in the scoring step.

### Component for atom signature generation

The molecule curation workers leave behind curated structures of molecule upon standardisation. Down the workflow, they are consumed by another worker that generates its atom signatures [9]. Atom signatures are structural descriptors – canonical, circular descriptions of an atom’s environment in a molecule. The atom signature of a given atom in a molecule is a directed acyclic graph of its connected atoms, where every node in the graph is an atom and the edges are the bonds between the atoms. The levels of neighbourhood of an atom in a molecule is the signature height of that atom. A molecular signature is the summation of all atom signatures of a molecule. The successful usage of molecular signatures is reported in various studies, ranging from QSAR calculations to prediction of enzyme-metabolite and target-drug interactions [9,10]. In their original implementation, Ertl *et al*[1] used HOSE codes, an earlier circular description of atom environments suggested by Bremser [11] for the use in NMR spectrum prediction. Atom signatures and HOSE codes capture identical circular description of an atom environment but only differ in their string representation. Since we had a well-tested, efficient implementation of signatures in the CDK, provided by Torrance [12], we decided to test whether it would give the expected identical results as the HOSE code-based implementation of the original work by Ertl *et al*[1]. The worker in the “Signature Scoring” folder generates atom signatures based on a given structure as input. The worker generates atom signatures of a molecule and tags them with the molecule’s UUID, to keep account of the signatures identity. The signature’s height (number of spheres in the atom environment used for signature generation) is configurable and we used atom signatures of height 2 (set as default) as it was sufficient in capturing relevant structural features in small molecules. The generated atom signatures for huge training datasets are usually written out to text file and stored for re-use. This feature is shown in Figure 1.

### Component for NP-likeness score calculation

**Figure 2:**
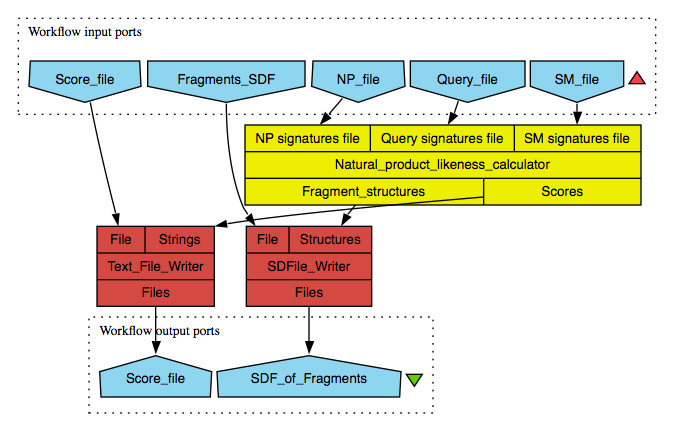
**NP-likeness scoring workflow.** This workflow takes input of atom signatures file of natural product (), synthetic (), and query () compounds dataset. The indexes the natural product and synthetic molecule signatures internally and generate NP-likeness scores for query compounds based on the presence or absence of its atom signatures in the index. The higher the score, the higher is the NP-likeness of the compound. The scores assigned with the corresponding compound UUID are written out to a text file. The UUID of the score can then be matched with the (Shown in Figure 1) to retrieve the full structure. The worker is helpful in visualising the distribution of compound scores in a dataset. The scorer worker also rebuilds fragment structure from the atom signature and assigns its corresponding fragment score as the fragment property. These fragment structures are written out to a SDF file as it is helpful in obtaining structures of high scoring fragments. The is an optional worker to visualise the re-built fragments from the atom signature. This workflow is available for free download at http://www.myexperiment.org/workﬂows/2121.html.

The worker in the “Signature Scoring” folder takes signatures of natural products, synthetic molecules and query compounds as input from text files. The workflow is depicted in Figure 2. Within this worker, atom signatures of compounds from Natural Products and Synthetic Molecules datasets are indexed separately, in order to look up for the frequency of molecule fragments in question. The number of atom signatures generated for a molecule is equal to the number of atoms that make up the molecule. Every atom signature independently represent a structural feature/fragment of the molecule, and an individual score for it is calculated using the statistic used in the original implementation.

(1) $$\mathrm{Fragmen}t_{i}=\log\left[\frac{NP_{i}}{SM_{i}}*\frac{SM_{t}}{NP_{t}}\right]$$

In the above calculation of single fragment contribution *Fragment*_*i*_, *NP*_*i*_ is the total number of molecules in the natural products dataset in which the *Fragment*_*i*_ occurs, *SM*_*i*_ is the total number of molecules in the synthetic molecules dataset in which the *Fragment*_*i*_ occurs, *SM*_*t*_ is the total number of molecules in the synthetic molecules dataset and *NP*_*t*_ is the total number of molecules in the natural product dataset. Individual fragment contributions from a molecule finally add up to give the total score of the molecule as shown in equation (2). The summed up score is then normalised by the number of the atoms in a molecule (N) as shown in equation (3), to give the final NP-likeness score for a molecule. Here, normalisation prevents molecules containing higher number of atoms from gaining higher score.

(2) $$\mathrm{Scor}e_{N}=\overset{N}{\underset{i=0}{\sum }}\mathrm{Fragmen}t_{i}$$

(3) $$\mathrm{NP}-\mathrm{likenessScore}=\frac{\mathrm{Scor}e_{N}}{N}$$

The calculated molecule scores are written out to a text file, tagged with their respective compound UUID. It is possible that non-linear discriminant analysis would work slightly better, but clear advantage of our approach is that it is chemically interpretable, it identifies fragments or substructures that play a role in Natural Product-likeness and this information may be used then directly in molecule design applications, for example for combinatorial library design or fragment growing. The nonlinear statistical methods provide mostly complex, not easy interpretable numerical solution. Further, natural product-likeness is a concept and there is no established standard of value range to compare against. worker, also under the “Signature Scoring” folder, makes density plots of the scores and writes it out in Portable Document Format (PDF). An example workflow making use of the scoring workers described above is shown in Figure 2.

## Results

The performance of the NP-likeness score depends, of course, on the choice of natural products and synthetic molecules in the training dataset. For the analysis of our engine’s performance, natural products, synthetic molecules and query compound collections were all obtained from open access databases only. Our first subset of natural products (22,876 molecules) originates from the ChEMBL database [13], where we selected molecules extracted from the *Journal of Natural Products*. The second subset of natural products (39,162 molecules) comes from the Traditional Chinese Medicine Database @ Taiwan (TCM)[14]. Together, the natural product training set comprised 58,018 non-redundant structures. Training set of synthetic molecules comprised 113,425 clean lead-like compounds selected from the ZINC database [15]. Small molecules from DrugBank [16] and the Human Metabolome Database (HMDB) [17] were treated as our test sets. Besides that, PubMed abstracts reporting isolation of new NPs were text-mined for natural product’s name and the names were converted into SMILES using Chemical Identifier Resolver [18] and the resultant set of 3610 non-redundant NPs was used as our test set.

**Figure 3:**
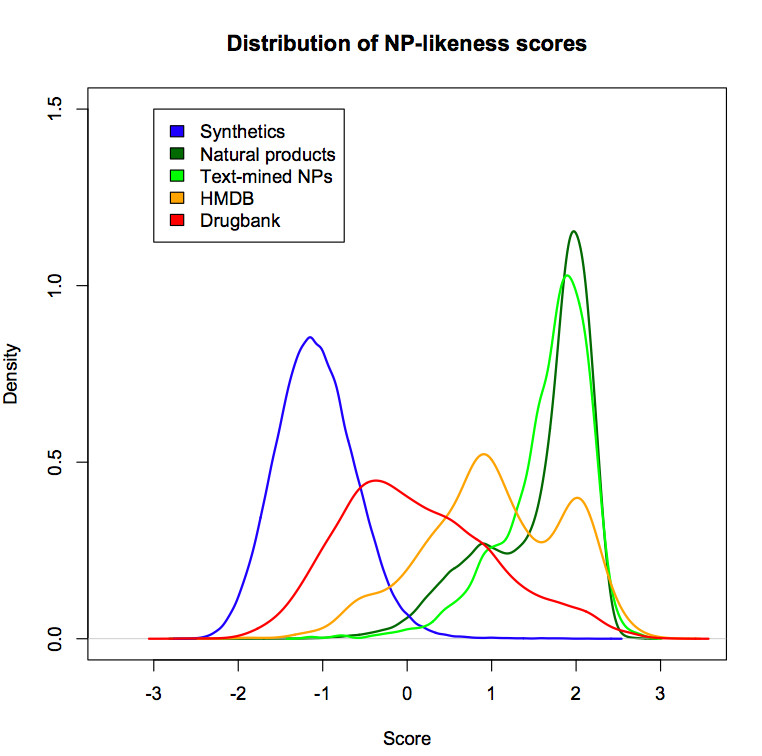
**Distribution of NP-likeness score for the training (synthetic molecules and natural products) and the test datasets.** The synthetic molecules are a subset of the clean lead-like collection from the ZINC database and the natural products are small molecules from ChEMBL database referenced to *Journal of natural products*. The more positive the score, the higher is the NP-likeness and vice versa.

The steps shown in Figure 1 were repeated for both training and test sets to calculate their atom signatures. To score test sets for NP-likeness, steps shown in Figure 2 were followed. The overall scores obtained in our test study ranged from -3 to +3. The more positive the score, the higher is the NP-likeness and vice versa. The distribution of scores obtained for the compounds in the test set is shown in Figure 3. The distribution of the DrugBank compound set overlaps both the synthetic molecule and natural product structural space. This is expected because, in drug design experiments, the drug-like compounds often end up mimicking structural features of metabolites after the optimisation process [19]. Only one third of the natural products space captured by us overlaps with currently available common drugs. The text-mined natural products, as expected, almost completely overlaps the training natural products structural space occupying small additional structural space.

To validate our scoring system, 3610 text-mined NPs with additional 5000 synthetics were scored using both our system and the original implementation by Ertl *et al*[1]. Despite the much larger training set of the original system, the scores obtained showed a good correlation coefficient with r-value 0.94. Further, the scores obtained for the test set by replacing the training data in the original system with our open-data, showed very good correlation coefficient with r-value 0.97. Taking into account that two cheminformatics toolkits that have been used to calculate the values, differ slightly in handling of aromaticity, tautomerism, molecule normalisation etc and also slightly different types of substructure fragments, we consider this agreement very good and fully validating the new implementation of NP-likeness.

## Conclusions

We have presented an open-source, open-data implementation of a Natural-Product-likeness scorer originally described by Ertl et al. Workflows for curation, training and scoring are implemented in the open-source workflow tool CDK-Taverna and published at myexperiment.org. A version of the scorer is available as an executable from command-line and as a library for inclusion in stand-alone or web applications. Training and test sets where extracted from open access databases such as ChEMBL, TCM, ZINC, DrugBank and HMDB. We replaced HOSE codes by Faulon’s atom signatures as our circular fingerprint implementation which showed similar performance. With the available open-data and open-source tool-kits, we have implemented a NP-likeness scorer engine and successfully demonstrated its capability to differentiate the natural product compound collection from synthetic and drug compound collections identical to what was reported in the original paper. The engine can be used as a filter to remove improbable metabolite structures from chemical spaces generated from Computer Assisted Structure Elucidation (CASE) or to select natural-product-like molecules from molecular libraries for the use as leads in drug discovery. The open-source, open-data implementation allows other researchers to modify the workflows or to use larger collections of training molecules once they become available.

## Competing interests

The authors declare that they have no competing interests.

## Authors’ contributions

PE, with his colleagues from Novartis, Basel, conceived the original idea of natural product likeness score. PE also provided the text-mined natural product dataset. CS conceived the idea of implementing the scoring engine using open source and open data. AT made new developments to the existing CDK-Taverna plug-in. KJ conducted the study, selected the data, implemented the curation, training and scoring engine and tested it. PM contributed to the development and discussion. All authors read and approved the final manuscript.
